# A life course approach to health and well-being: call for papers

**DOI:** 10.2471/BLT.26.296161

**Published:** 2026-05-01

**Authors:** Ritu Sadana, Vanessa De Rubeis, Chandni Maria Jacob, Janeen Baxter, Yoav Ben-Shlomo, Neal Halfon, Monique Verschuren

**Affiliations:** a Department of Sexual, Reproductive, Maternal, Newborn, Child, Adolescent Health and Ageing (Life Course Health and Research), World Health Organization, Avenue Appia 20, 1211 Geneva 27, Switzerland.; b ARC Centre of Excellence for Children and Families over the Life Course, Institute for Social Science Research, The University of Queensland, Brisbane, Australia.; c Population Health Sciences, Bristol Medical School, Bristol, England.; d Department of Pediatrics, UCLA Geffen School of Medicine, Los Angeles, United States of America.; e Department of Lifecourse, Lifestyle and Health, National Institute for Public Health and the Environment, Bilthoven, Kingdom of the Netherlands.

Health and well-being are shaped by the interaction of biological, psychosocial and environmental determinants acting across the entire lifespan and between generations. A life course approach recognizes the cumulative effects of these factors during sensitive and critical periods and key transitions across life stages,^1^^,^^2^ as well as the importance of context and locally adapted health systems.^3^ Addressing this continuum requires health systems designed not merely to treat disease but also to optimize physical and mental health trajectories, from birth and early childhood through adolescence and adulthood, to healthy ageing and a dignified death.^4^

The life course approach is becoming increasingly prominent in global and regional health policy frameworks for strengthening systems and promoting health and well-being across generations.^5^^,^^6^ The World Health Organization’s (WHO) *Framework to implement a life course approach in practice*^7^ establishes the normative and operational foundation for this reorientation. The framework articulates six core principles: (i) focus on the whole person through a comprehensive person-centred approach to strengthening health capacities; (ii) promote health equity; (iii) enable early action in each life stage; (iv) put in place evidence-based action; (v) ensure actions are taken together across sectors and generations; and (vi) ensure actions cover and connect all life stages and critical periods.^7^ The framework highlights how structural determinants (that is, natural environment, power structures, genetic inheritance and social position) may operate through intermediary pathways at the individual, household and community levels through positive or negative exposures or vulnerabilities. The framework also accounts for how these structural and intermediary determinants interact with proximal determinants such as systems (health, education, labour or social security), services (including public goods and commercial), infrastructure (including built environment) and people’s lived experiences that together shape health trajectories. Based on existing evidence, strategic actions including in health and other sectors, financing, stewardship and the use of data on each life stage, especially longitudinal and inclusive of older adults, can be used to reorient actions aligned with the six principles, at scale.

However, disease-focused and vertically organized programmes continue to dominate health systems, limiting integration of life course principles across life stages. By strengthening the knowledge, capacity and resources to operationalize the approach across diverse settings such as low- and middle-income countries, primary to tertiary services, in disasters and conflicts, health systems can be reoriented towards population needs and optimizing health capacities.^8^^–^^10^ The life course approach also offers a strategic lens for advancing universal health coverage towards evolving population needs within the context of demographic change. When embedded within primary health care and the delivery of universal health coverage benefit packages, the approach can support essential service delivery during critical periods and within each life stage to foster health benefits and social and economic development.^11^

The *Bulletin of the World Health Organization* calls for papers inspired by the WHO framework and its evidence and implementation priorities. Articles should address how to put a life course approach into practice, what works at the population, individual and/or group level, and how effectiveness is assessed. We welcome papers on implementation across all levels of health-care provision and approaches to coordinate and offer continuity of care, inclusive of families and carers. We specifically encourage papers on approaches to optimize, maintain and track physical and mental health capacities across the life course; the benefits and ways to incorporate a life course approach within disease, condition or intervention-specific programmes such as diabetes, obesity, sexual and reproductive health or immunizations; to connect actions across the life course through population-based interventions that benefit everyone; as well as to implement targeted interventions to prevent or mitigate the impact of negative exposures or conditions at each life stage.

Submissions may address strategy, policy and applications within one or multiple countries; comprehensive approaches to adult health and well-being; use of longitudinal data and life course modelling approaches that identify opportunities for action; analyses that identify critical periods and leverage points for interventions; or financing and governance mechanisms that enable multisectoral implementation and a whole-of-government approach to domestic resource allocation. We particularly encourage submissions from and about low- and middle-income settings, and from multidisciplinary and cross-regional teams.

All types of articles are welcome and manuscripts should be submitted in accordance with the *Bulletin*’s guidelines for contributors^12^ by 1 August 2026, and the cover letter should mention this call for papers.
